# Building Data-Driven Pathways From Routinely Collected Hospital Data: A Case Study on Prostate Cancer

**DOI:** 10.2196/medinform.4221

**Published:** 2015-07-10

**Authors:** Joao H Bettencourt-Silva, Jeremy Clark, Colin S Cooper, Robert Mills, Victor J Rayward-Smith, Beatriz de la Iglesia

**Affiliations:** ^1^ School of Computing Sciences University of East Anglia Norwich United Kingdom; ^2^ Norfolk & Norwich University Hospital NHS Foundation Trust Norwich United Kingdom; ^3^ School of Biological Sciences University of East Anglia Norwich United Kingdom

**Keywords:** hospital information systems, data summarization, clinical pathways, data quality, visualization, prostate cancer, electronic medical records

## Abstract

**Background:**

Routinely collected data in hospitals is complex, typically heterogeneous, and scattered across multiple Hospital Information Systems (HIS). This big data, created as a byproduct of health care activities, has the potential to provide a better understanding of diseases, unearth hidden patterns, and improve services and cost. The extent and uses of such data rely on its quality, which is not consistently checked, nor fully understood. Nevertheless, using routine data for the construction of data-driven clinical pathways, describing processes and trends, is a key topic receiving increasing attention in the literature. Traditional algorithms do not cope well with unstructured processes or data, and do not produce clinically meaningful visualizations. Supporting systems that provide additional information, context, and quality assurance inspection are needed.

**Objective:**

The objective of the study is to explore how routine hospital data can be used to develop data-driven pathways that describe the journeys that patients take through care, and their potential uses in biomedical research; it proposes a framework for the construction, quality assessment, and visualization of patient pathways for clinical studies and decision support using a case study on prostate cancer.

**Methods:**

Data pertaining to prostate cancer patients were extracted from a large UK hospital from eight different HIS, validated, and complemented with information from the local cancer registry. Data-driven pathways were built for each of the 1904 patients and an expert knowledge base, containing rules on the prostate cancer biomarker, was used to assess the completeness and utility of the pathways for a specific clinical study. Software components were built to provide meaningful visualizations for the constructed pathways.

**Results:**

The proposed framework and pathway formalism enable the summarization, visualization, and querying of complex patient-centric clinical information, as well as the computation of quality indicators and dimensions. A novel graphical representation of the pathways allows the synthesis of such information.

**Conclusions:**

Clinical pathways built from routinely collected hospital data can unearth information about patients and diseases that may otherwise be unavailable or overlooked in hospitals. Data-driven clinical pathways allow for heterogeneous data (ie, semistructured and unstructured data) to be collated over a unified data model and for data quality dimensions to be assessed. This work has enabled further research on prostate cancer and its biomarkers, and on the development and application of methods to mine, compare, analyze, and visualize pathways constructed from routine data. This is an important development for the reuse of big data in hospitals.

##  Introduction

### Clinical Pathways

Clinical pathways, also known as care or critical pathways, have been introduced in health care systems to improve the efficiency of care, while maintaining or improving its quality [1]. In 1995, Pearson et al [2] described critical pathways as a management plan that “displays goals for patients and provides the sequence and timing of actions necessary to achieve these goals with optimal efficiency”. More recently they have been described as a concept for making patient centered care operational, and for “supporting the modelling of patient groups with different levels of predictability” [1]. Clinical pathways are developed by multidisciplinary teams and rely on evidence from the literature, operational research, and patient involvement methodologies [1].

Over the years, pathways evolved from paper-based to computerized pathways [3,4], and there have been efforts to integrate them with electronic health records [4,5]. The support for guidelines and pathways is one of the most promising fields for knowledge-based systems in health care [6]. The standard functions of pathways have been proposed in [4], and a strong emphasis is given to the statistics function to implement automated methods for checking the occurrence of variance (ie, discrepancies between planned and observed events).

There are several definitions of clinical pathways in the literature, but in this paper they are defined as an ordered set of patient-centric events and information relevant to a particular clinical condition. In this paper, a clinical pathway is not described in the context of an intervention, but in the context of the description, analysis, and evaluation of clinical parameters for a specific condition over time. The pathways are also data driven and allow the inspection of routine hospital data that would otherwise be overlooked. Furthermore, we place particular importance on the use of clinical biomarkers and other indicators (such as blood readings) in pathways, as they enable a thorough inspection of data quality, as well as further clinical studies observing trends over time.

### Analysis of Clinical Pathways

The analysis of clinical pathways is a topic receiving increasing attention in medical informatics, but techniques often require extensive clinical expert knowledge and can be laborious. Huang and Duan [7] used process mining techniques to measure clinical behavior derived from clinical workflow logs and to help identify novel process patterns. According to them, clinical pathway analysis has been defined as the process of discovering knowledge about clinical activities in patients’ care journeys. Ultimately the goal is to utilize the discovered knowledge for pathway (re)design, optimization, decision support, audit, or management, and one of the major challenges reported was the derivation of compact, yet high quality, patterns that cover the most useful medical behaviors in clinical practice.

Process mining techniques are promising analysis techniques in the context of clinical pathways. However, it has been reported that traditional process mining algorithms do not cope well with unstructured processes like those commonly found in a hospital environment [8,9], and that they may not produce clinically meaningful visualizations. The heterogeneity and incompleteness of the data are major obstacles in achieving meaningful models, yet an application to stroke has proved fruitful [10]. An aim of this paper is to produce pathways that may be suitable for process mining. For this, data quality is key, but consensus and definitions are lacking [11,12], and intelligent agents that explore quality issues are needed [12].

The use of routine data or workflow logs in the construction of clinical pathways is a key topic receiving increasing attention in the literature [7-9]. In hospitals, such efforts rely heavily on the hospital information systems (HIS) and electronic health records (EHR), and the availability and quality of the information conveyed in them. Indeed, hospitals often opt for implementing several commercial departmental systems, creating "islands" of information across various departments [13,14]. This can significantly hinder the process of extraction and collation of detailed patient-centric information to create clinical pathways. The methods presented in this paper attempt to overcome some of these difficulties.

### Data Quality in Electronic Health Records

A review on data quality in EHR [11] identified five data quality dimensions described in the literature: (1) completeness, (2) correctness, (3) concordance, (4) plausibility, and (5) currency. However, the authors identified that not all dimensions are commonly or consistently assessed, and further work is needed toward the adoption of systematic, statistically based methods of data quality assessment. The work presented in this paper enables the inspection of data quality dimensions with a particular emphasis on assessing the completeness of pathway information using biomarker expert rules.

Overall, this paper describes a framework for building and visualizing prostate cancer pathways using routinely collected data from a large United Kingdom National Health Service (NHS) hospital. This approach does not involve workflow logs produced by HIS or EHR, but rather, the patient-centric data conveyed in them. Our previous work on methods for the collection of patient-centric data from multiple HIS [14] has underpinned this research.

### Prostate Cancer

The latest estimates of global incidence indicate that prostate cancer has become the second most common cancer in men [15]. In the United Kingdom, it is the most common male cancer, accounting for 25% of all malignancies [16]. In recent years, there has been a generalized increase in reported incidence, but, despite this, the mortality rates have been on the decline [16-18]. Nevertheless, the economic burden of prostate cancer will continue to rise due to increased diagnosis, diagnosis at an earlier stage, and prolonged survival [18]. It has been reported that new strategies need to be devised to increase the efficiency of health care provision for this type of cancer in order to tackle the increasing burden [18]. Prostate Specific Antigen (PSA), a biochemical marker used clinically for prostate cancer detection and prognosis, is associated with substantial overdiagnosis and excessive treatment [19], which makes its utility as a screening test controversial, and warrants the need for further studies.

The National Institute for Clinical Excellence (NICE) in the United Kingdom publishes clinical guidelines and has recently developed the NICE pathways, a tool that visually represents the recommendations and guidelines on a specific clinical or health topic [20]. Following the NICE pathway, patients with suspected prostate cancer are directed through from referral, to assessment, diagnosis, and communication; their needs are then often discussed at a multidisciplinary team meeting; admission and treatment options are selected as appropriate, and ultimately patients are followed up, and outcomes assessed. During each step of the pathway, relevant patient-centric data are produced and often stored in a variety of different HIS. Clinicians wishing to investigate prostate cancer, say to establish the merits of alternative treatment and management options, would have a powerful tool if access to the integrated data was facilitated in an electronic and canonical form. However, as is often the case with HIS, database systems and their data are heterogeneous, and data quality, accessibility, and interface vary considerably.

### Objectives

The aims of the work presented here can be divided into two: (1) to generate individual data-driven patient-centric pathways from routinely collected hospital data for prostate cancer, and (2) to evaluate the completeness and utility of the generated pathways for investigating biomarker trends. The latter allows for the selection of high quality data for clinical studies and decision making, which, in turn, enables the (re)design, management, and optimization of pathways. We focus on a definition of a pathway as a data structure that synthesises knowledge, and facilitates the development of methods for the computation of variance and other statistics. The framework presented in this paper, together with their formalisms, should allow and encourage other tools and techniques, such as process mining or ad-hoc algorithms to be used.

##  Methods

### Prostate Cancer Case Study

A case study on prostate cancer was carried out at the Norfolk & Norwich University Hospital (NNUH) NHS Foundation Trust with data from this hospital only. Appropriate credentials were obtained from the National Research Ethics Service (Norfolk) and NNUH research governance committees, and no patient consent was required. The data were anonymized and no patient sensitive information such as names or addresses was used.

This section first summarizes the methods for data collection from multiple hospital sources under the subheading “The Operational Data Store”, and it is followed by the definition of a pathway under the subheading “Extraction of the Study Datasets”. Descriptions of the methods to build a pathway dictionary and to generate the pathways are given under the subheading “Building the Pathway Dictionary and Database”. The subsection “Visualizing Pathways and Overall System Architecture” introduces the system to integrate, visualize, and analyze the pathways, as well as a novel graphical representation, and the subsection “Assessing Completeness Using Biomarker Information” describes a method for assessing the quality of the pathways.

###  The Operational Data Store

Electronic patient data in hospitals are usually complex and heterogeneous [21,22], scattered through several information sources or HIS, and its retrieval methods are often ad-hoc and poorly described in the literature [14,23]. A previously proposed data extraction process [14] was used to collect patient-centric data from HIS, and it is summarized in this section.

**Figure 1:**
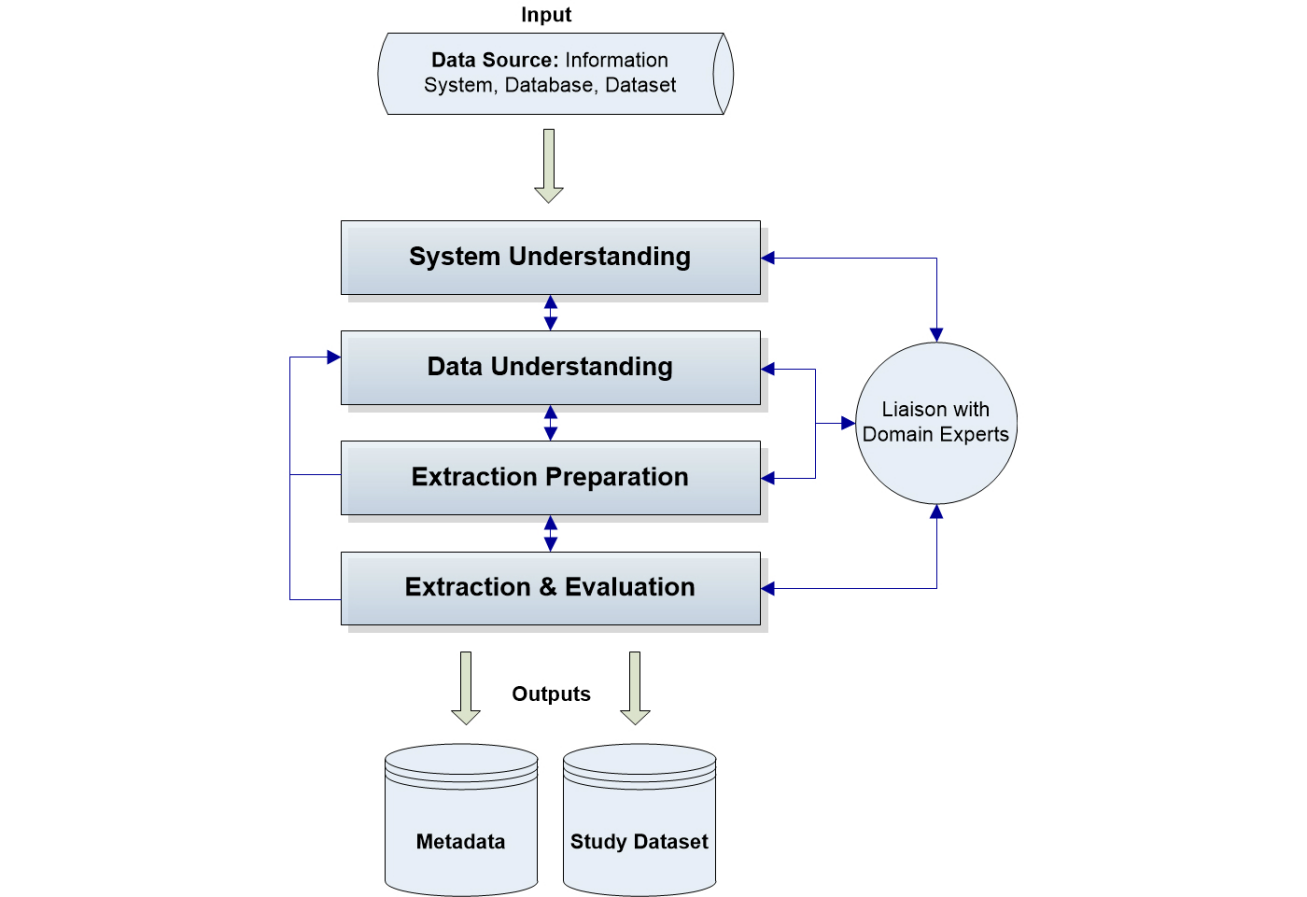
Simplification of the data extraction process [14].

The process involves liaising with domain experts (or subject matter experts) to identify data sources where information related to prostate cancer patients is likely to be stored (eg, radiology). In this case study, the team of experts included a urology consultant, prostate cancer geneticists, a consultant oncologist, a histopathologist, and a chemical pathologist. For each data source identified (a EHR or HIS), the data extraction process [14] was followed. The process consists of four key steps and Figure 1 shows this: (1) system understanding, where each data source is investigated and details about the system are gathered; (2) data understanding, where data familiarization, selection, and building the data dictionary occurs; (3) extraction preparation, where data extraction methods are prepared or reviewed; and (4) extraction and evaluation, where data are extracted, validated, and the process is evaluated.

An example of an input data source is the laboratory information system (LAB), where information on the PSA and other blood tests are stored. Following the data extraction process in Figure 1, a thorough inspection of the system is carried out first (system understanding step). This required the involvement of domain experts (clinical and administrative), obtaining relevant access credentials and previewing the system, and resulted in an understanding of the way in which blood tests are requested and how that information flows in and out of the LAB system. The next step deals with understanding the data. In this example, data on PSA were explored, including details on how it had been recorded over time, data field semantics, and available patient and blood test identifiers that, for example, allow the retrieval of unique blood tests for each patient. Once both systems and data were investigated with respect to the required information (in this example, the PSA), then a suitable data extraction strategy is devised. Finally, the selected methods are tested to ensure that they produce the same desired results. In this example, the LAB system offered an on-line analytical processing interface, where additional training and input from domain experts was required in order to produce database queries that retrieved the PSA test data along with dates, times, and identifiers for data linkage purposes. Sample datasets are extracted in a suitable format, and subsequently they are evaluated. The evaluation consists of cross-checks against the LAB system and patient notes, and a careful examination for missing or erroneous values (for example, nonnumeric values were identified in some of the PSA test results: <0.1 ng/ml). Erroneous values are corrected when possible (for example, <0.1 was reformatted to 0.05) or their records are eliminated. Finally, a study dataset is produced for the LAB system containing the PSA tests. A second output is metadata (about the source, its tables, attributes, and values) that is generated at each step of the process, and allows it to be repeated and documented over time.

The process is repeated for every data source where information on prostate cancers is likely to exist, and this ultimately generates an operational data store (ODS), which is similar to a data warehouse from where specific data marts can be extracted. The ODS contains relevant metadata and detailed, routinely collected information on the selected case study. By enabling the inspection, linkage, and compilation of cohorts, it helps to overcome the types of heterogeneity commonly found between HIS such as technical differences, syntactic, and semantic heterogeneity. This process is also suitable for, and greatly facilitated in, less heterogeneous environments where data sharing standards exist.

Overall, the data extraction process enables the use of routinely collected data to build a repository containing all interactions of the patient with the hospital. This process can be repeated so that the ODS continues to be populated with new records. The methods of extraction are reviewed and revised over time. The costs associated with this process depend on the functionalities of the HIS, particularly with respect to the retrieval of cohorts of patients, as well as documentation and support. The process may be time consuming in systems where no querying tools are available, and alternative methods are required. Overall, the most time consuming step of the process, given our experience in this case study, was system understanding, where a substantial amount of time was spent liaising with hospital information technology managers and other staff, and the second most time consuming step was extraction preparation. However, after the first iteration, the process becomes streamlined and only minor adjustments may be required even in heterogeneous environments. The process is also applicable to more structured environments where reduced costs are to be expected. Different problem domains are not expected to require other costs, as these are mostly dependent on the HIS rather than on particular data elements.

The data available in the ODS may be more than required for a particular clinical study, as the retrieval process is based on minimum use of constraints. However, this provides a holistic representation of the patients, including their demographics, comorbidities, test results, or other information, and is limited by the availability of electronic information in the HIS. The selection of specific data elements from the ODS that will form a pathway is performed later (“Building the Pathway Dictionary and Database”) in consultation with the domain experts. A summary of the data retrieved from the ODS for creating pathways is given in the Results section.

###  Extraction of the Study Datasets

In the case study on prostate cancer, the ODS contains information from the following systems: administration, cancer waiting times, histopathology, radiology, biochemistry, operating theater, orthopaedics, oncology, and radiotherapy. However, not all sources are used in the pathways presented in this paper, as later explained in “Building the Pathway Dictionary and Database”. Table 1 shows the data sources used in the development of the pathways. Retrieving diagnosis codes from the administration and histopathology systems first identified the prostate cancer cohort, and it was later validated with information from the local cancer registry.

**Table 1:** Data sources used for the development of the pathways.

Data source (abbreviation)	Description of selected data
Administration (ADM)	Patient episodic information, comorbidities, and clinical coding.
Histopathology (HIST)	Histopathology reports and extracted Gleason grades.
Radiology (RAD)	Radiological imaging limited by reports where the word prostate occurs.
Biochemistry (LAB)	PSA tests. However, other blood tests can be added.
Operating theater (OT)	Operating theater procedures and coding.
Radiotherapy (RT)	Radiotherapy treatments dates and number of sessions.
Cancer registry datasets (CR)	The cancer registry dataset includes some of the above data, which can be used for quality checking purposes, and additional data such as cause of death.

### The Prostate Cancer Cohort

For the prostate study, a cohort of 1904 patients diagnosed with prostate cancer (average age 72, SD 9) between 2004 and 2010 was selected for retrieval from the ODS. This represents a subset of the total number of prostate cancers, where it was possible to accurately ascertain both diagnosis and treatment dates. Ascertainment of nearly 20% of the original cohort was not possible due to the information not being consistently recorded, to changes in systems and the way they are used, and to data quality not being consistently inspected prior to 2008. Data from 2003 were collected and used as potential “screening” and from 2011 as follow-up. This time window delimits patient pathways. Date and cause of death were collected from the cancer registry early in 2012. All patients in the cohort have a diagnosis date and have been offered treatment as per the UK guidelines. The UK national cancer waiting time guidelines stipulate that all suspected cancers in the NHS should be offered treatment (including active surveillance) within 31 or 62 days, according to the national cancer waiting times guidelines. As per the cancer waiting times guidelines, all patients were followed up after diagnosis and, in this cohort, 2.21% (42/1904) did not agree on any form of treatment. This differs from active surveillance, in that the latter requires the patient and clinician to agree to monitor tumor growth.

Additional information not consistently recorded in HIS (eg, tumor staging) was retrieved from the local cancer registry (CR) using deterministic record linkage on national health identifiers and dates of birth. The registry served as a source of validation for the collected data as most of the critical data elements often used in prostate cancer studies will be present in the local CR. However, additional hospital data that were routinely collected, but not present in national audit reports or cancer registries (such as biomarker trends or imaging) increases the value and completeness of the pathways. In particular, the value of the biomarker in determining the quality of the pathways is discussed later in this paper.

###  Defining a Pathway

In order to create pathways, data elements are selected from the ODS and its sources (Table 1). A formal definition of a pathway is given in the “Definitions” subsection, and further details on the selection of data elements and their inclusion in a pathway data dictionary are given in the “Building the Pathway Dictionary and Database” subsection. The developed software environment, data flows, and visualizations are described in the subsection “Visualizing Pathways and Overall System Architecture”, and the proposed methods to compute completeness based on biomarker elements within a pathway are given in the subsection “Assessing Completeness Using Biomarker Information”.

###  Definitions

Let D represent the pathway dictionary, where the *i*-th entry has a code *c*_*i*_ *(1≤i≤n)* in a total of *n* possible codes described in detail in Table 2. *C*_*E*_ is the subset of codes containing timed events, and *C*_*I*_ the subset containing informational elements, such as demographics. By associating a zero time with informational elements, all events in the pathway can be viewed as timed events.

A *pathway activity* A is then defined as four-tuple *A*=(*r,t,c,v*) where,

- *r* is the patient identifier
- *c* ∈ *C* is an event code
- *t* is the time in days before or since the day of diagnosis recorded for patient *r*
- *v* is a value, numerical or categorical, associated with dictionary code *c*

A *pathway* for patient, *r*, is represented as a chronological sequence of activities, *P=*(*A*_*1*_ *,A*_*2*_ *,...,A*_*m*_), where

1. *A*_*i*_ is of the form (*r, t*_*i*_ *, c*_*i*_ *, v*_*i*_) for 1≤*i*≤*m*,
2. *t_i_≤t_i_* +1 for 1*≤i≤m,*
3. any *A*_*i*_ with *c* ∈ *C*_*I*_ has *t*_*i*_=0,
4. if *A*_*i*_ = (*r, t*_*i*_ *, c*_*i*_ *, v*_*i*_) and *A*_*i+1*_ = (*r, t*_*i+1*_ *, c*_*i+1*_ *, v*_*i+1*_), then there is no activity *A* = (*r, t, c, v*) where
5. *t*_*i*_< *t* *<* *t*_*i+1*_, and
6. all relevant activities involving patient *r* appear in *P.*

Note that when *t*_*i*_=*t*_*i+1*_ for 1≤*i*≤*m*-1, the corresponding activities *A*_*i*_ and *A*_*i+1*_ are concurrent.

A simple pathway for patient *r=1* might be *P*=〈 *A*_*1*_=(1,-28,P,45), *A*_*2*_=(1,0,D,2), *A*_*3*_=(1,1,G,"4+3"), *A*_*4*_=(1,1,H,"Cyproterone Acetate"), *A*_*5*_=(1,151,R,"37"), *A*_*6*_=(1,260,P,0.2), *A*_*7*_=(1,340,P,0.05), *A*_*8*_=(1,539,P,0.05) 〉.

In this patient’s pathway, the first PSA test was elevated at 45 ng/ml, and this led to the diagnosis of stage 2 prostate cancer, with a Gleason grade of 4+3. Note that the biopsy was performed as an outpatient event, and hence, it is unavailable in this pathway, however, the histopathological findings of that biopsy are present. The patient then agreed to undergo hormone therapy (cyproterone acetate) and a subsequent 37 sessions of radiotherapy. The number of radiotherapy sessions is recorded as value of element code *R*. Information on specific sessions was not consistently available at the time, and was therefore not used. The radiotherapy sessions were then followed by PSA readings of 0.2 ng/ml and two readings <0.1 ng/ml, which indicate a good response to treatment.

The above model of expressing pathway activities is similar to the entity-attribute-value (EAV) data model [24], where concepts are described in an attribute in a row. Later, the i2b2 data model [25] expanded on the EAV model to account for time (start and end dates for each observation). This, together with a star schema, has been described as an extremely efficient way of querying data, as a large index can be built to encompass all patients' data in the master table [25]. The proposed pathways model expands the EAV model in that every row has an associated time, and this is important because pathways are ordered sets of events. With regards to the i2b2 model, the proposed pathways include fewer elements in the master table, and focus on a sequential representation and processing of pathway activities. In addition, activities and their pathways can also be linked to other tables (and dimensions) that store other types of information, similarly to what is accomplished by the star schema in the i2b2 model. The proposed pathways model is part of an overall framework environment that is described in detail in the subsection “Visualizing Pathways and Overall System Architecture”.

**Table 2:** Pathway dictionary for prostate cancer.

Class	Code	Name	Type	Data source	Frequency, n (%)
Demographics	Q	Deprivation score	Information	CR^a^	1904/1904 (100.00)
Demographics	A	Age at diagnosis	Information	CR^a^	1904/1904 (100.00)
Demographics	Z	Death	Event	CR^a^+ODS (ADM^b^)	402/1904 (21.11)
Demographics	L	Clinical trial	Information	ODS (ADM^b^)	22/1904 (1.16)
Demographics	X	Other cancers	Event	CR^a^	406/1904 (21.32)
Diagnostics	D	Diagnosis and staging	Event	CR^a^+ODS (HIST^c^+ADM^b^)	1904/1904 (100.00)
Diagnostics	G	Histology grade	Event	CR^a^+ODS (HIST^c^)	1609/1904 (84.51)
Diagnostics	I	Imaging	Event	ODS (RAD^d^)	291/1904 (15.28)
Diagnostics	P	PSA test	Event	ODS (LAB^g^)	1814/1904 (95.27)
Treatment	S	Surgery	Event	CR^a^+ODS (OT^e^)	640/1904 (33.61)
Treatment	R	Radiotherapy	Event	CR^a^+ODS (RT^f^)	395/1904 (20.75)
Treatment	C	Chemotherapy	Event	CR^a^+ODS (ADM^b^)	8/1904 (0.42)
Treatment	O	Orchidectomy	Event	CR^a^+ODS (OT^e^)	2/1904 (0.11)
Treatment	H	Hormone	Event	CR^a^+ODS (ADM^b^)	960/1904 (50.42)
Treatment	W	Active surveillance	Event	CR^a^+ODS (ADM^b^)	422/1904 (22.16)
Treatment	N	No treatment	Information	CR^a^+ODS (ADM^b^)	42/1904 (2.21)

^a^CR = Cancer Registry datasets

^b^ADM = administration

^c^HIST = histopathology

^d^RAD = radiology

^e^OT = operating theater

^f^RT = radiotherapy

^g^LAB = biochemistry

###  Building the Pathway Dictionary and Database

**Figure 2:**
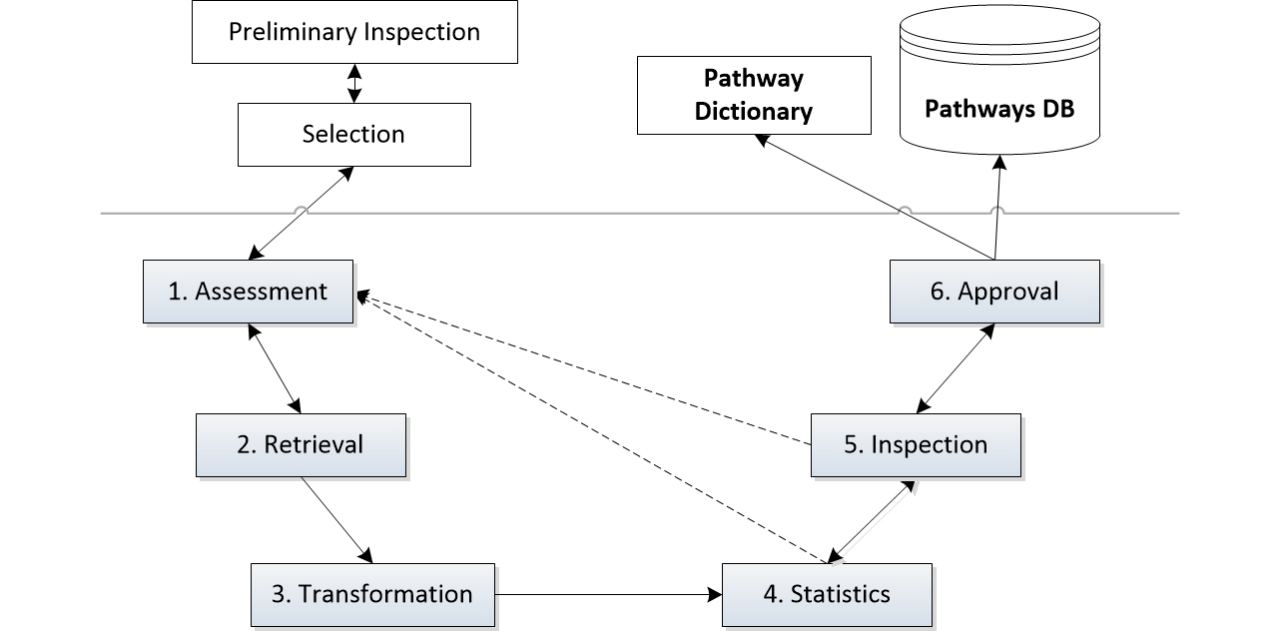
Methodology to build pathways’ dictionary and database (DB).

The selection of key informational requirements for the pathways is facilitated by the patient-centric approach to data collection [14]. The ODS contains data from the retrieved hospital sources and metadata, which allows for the inspection, linkage, and integration of semantic and syntactically different data. Nevertheless, the ODS may contain information outside the domain of a specific pathway. Therefore, in order to build a pathway dictionary, it is crucial to identify, select, and retrieve key data elements. Figure 2 illustrates the process of building a pathway dictionary from the data in the ODS, and is inspired by the similar data warehousing technique of extract-transform-load [26]. The pathway dictionary can be regarded as a simple ontological knowledge base, built by a bottom-up process, from available data to concepts. Temporal ontologies have been developed [27], yet for the definition of pathways, the above time-oriented data structure together with a pathway dictionary was sufficient to enable temporal abstractions.

The dictionary building process, based on input from domain experts, literature survey, and current prostate cancer guidelines, involves gathering relevant data elements and applying transformations to either create new features or strip out irrelevant elements (eg, hospital events that are neither exclusive nor relevant to the treatment of prostate cancer). At the end of this process, and for each data element, a flat file with the data corresponding to that element is created in the four-tuple transactional format described in the subsection “Definitions”. The steps involved in this process are described in detail below. At present, the system has not used multimedia or other large files, but plans are underway to ensure that such files can be encrypted and stored locally. In such cases a pointer to the file would be included in the relevant data element.

### Preliminary Inspection and Selection

The domain experts collaborate on a first inspection of the available data in the ODS to help with the identification of key data elements to be included in the pathways. This involves examining summary statistics (such as frequencies of biochemistry tests or other descriptive statistics) and metadata (such as attributes descriptions, semantics, or expected outliers) from the ODS, and is important as it sets the granularity of the pathways and the extent to which they can be meaningful for a particular disease. This process was mostly ad-hoc, as each data element required different statistics, and was also based on contributions by the domain experts. Data updates, however, may be processed automatically once the pathway dictionary is built.

For the prostate cancer study, we defined three classes of information: demographics, diagnostics (including investigations), and treatment. Hence, the selected elements in this step have an associated class. Further to this, each element type can either be a timed event, describing a particular activity at a given time, or auxiliary information, such as demographic data or other nonevent data, such as a patient’s participation in a clinical trial. Both class and type are two properties common to all elements of the pathway, and can be determined a priori or throughout the process of building the pathways as explained below.

For each selected data element, the following six steps are carried out to create a complete pathway dataset and dictionary. Throughout the following steps, we will use the example of the biomarker test for prostate cancer (PSA test) as a data element.

### Assessment

The first step is to inspect the element’s values as well as its semantics, syntax, and data type, and any potential limitations that may interfere with the consistency of the data element. Additional mapping, linkage, and transformations may be necessary to enforce a consistent format and these should be identified here. An example arising from the PSA test was the need for the removal of values that include symbols, such as “<1”, meaning the PSA test value is less than one. In this case, such values were replaced by 0.5. A first classification of the element is also given by assigning a dictionary code and the element type (informational or timed event); in this case the code for the timed event PSA test is “P”.

### Retrieval

The set of attributes and values for the data element are retrieved from the ODS. In the case of the PSA test, the attributes in the ODS include dates of test authorization, date of entry, value, comments, clinical history, fasting, blood reading thresholds, and the patient identifiers. More complex data elements, such as social determinants can also be created from the information available in the ODS, but they might require additional or specific preprocessing. For example, in this case study, deprivation score was included in the pathways (code Q in Table 2), and regarded as an informational element. Because of the way in which the deprivation index was recorded, the data element was in this case time-independent, and handled differently by the pathways framework described in the next section.

When retrieving information to create or update a data element, rule-based deterministic record linkage can be used to enforce constraints. In the course of the case study, the retrieval step was used to select data within the study time period as well as validating data from the hospital sources against the cancer register datasets, where possible, in terms of completeness, correctness, and concordance. The retrieved attributes must have the information required by pathway definition. The data for the particular element are then stored, and in this case study a comma separated file (CSV) is created to this effect. For PSA tests, the attributes selected from the ODS to be included in the pathway were date of entry (date when the sample was taken from the patient within the selected time period), the value, and the identifier that allows linkage. Working with CSVs can introduce additional technical challenges, in particular when different database or spreadsheet systems are used. In this case study, the data available in the ODS were extracted in a format that is compatible with CSV. However, additional checks using raw text editors and spreadsheet software may need to be performed after the data are extracted, so as to inspect and ensure that the exported data meet the required CSV constraints.

### Transformation

The retrieved data file is converted into the pathway data structure, with attributes *Identifier, Code, Date* (instead of time)*,* and *Value*, where *Code* is a constant. *Date* is used here, but it will later be converted into time, *t*, zeroed at diagnosis date. The latter, by removing full dates, allows an additional layer of anonymity to the pathways, as well as a basis for comparison among patients. Any necessary transformations and formatting changes identified in the previous steps are undertaken here.

### Summary Statistics

Summary statistics are produced in this step. These include distributions of the *Value* attribute, which can help to detect potential bias, together with overall support (ie, total number of patients); value-specific support (ie, number of patients on each value category); and extremes. Such statistics may help to detect and correct quality issues by assessing completeness (missing data), correctness, and plausibility. Additionally, other statistics may be produced, such as the number of values within a range; this is particularly useful for producing a summary of abnormal blood readings, such as raised PSA tests.

### Inspection

Together with the domain experts, the retrieved data and descriptive statistics are inspected. The values of the attributes are also checked for format consistency and the quality dimensions described above. At this stage, a decision regarding the data element is reached. The element may be:

- Kept as is, should it contain sufficient information and adequate support;
- Rejected, because there may not be enough information or support, or because the formats or data types no longer match those previously collected. The latter may lead to a reevaluation of the methods used to extract the data. However, this is not expected to happen when the process in Figure 1 is followed, and consistent metadata is also collected; and
- Subject to decomposition, into two or more elements, should the values of the element vary qualitatively, creating a source of ambiguous information, or should the requirements of a particular study involve inspecting a particular quantitative range, such as the abnormal range of a blood reading.

In the example of the PSA test, the data element was kept after the values were set to a canonical form.

An example of an element that was rejected in the case study is *biopsy*, because of insufficient support (this is further discussed below). A further example of an element that was split was *surgery*, into *orchidectomy* and *surgery* (prostatectomy). Another example of an element that was split was *radiotherapy*, where, for the analysis of the trend of PSA, only *radical radiotherapy* was interesting to investigate, as it affects the PSA.

### Approval and Update

Upon inspection, a decision is made regarding the data element and its values. When the decision is favorable, an update is carried out. The update is concerned with the technical work of merging the table containing the data element and its values with the pathways database master table. Further transformations are also carried out to sort the master table by date and patient identifier, and to compute time *t* zeroed at diagnosis date. This can be achieved by either creating an informational element providing the date of diagnosis or by programmatically isolating the specific date from an existing element and subsequently setting *t* for all activities in a pathway. The pathways dictionary is then updated with summary information.

The process of building the pathways data dictionary can be revisited to accommodate new data or to change the way in which informational elements are modelled. For example, should informational elements later be provided with a time-point, these can be remodelled as timed events and instructions can be added so that the software framework handles them differently. The latter is described in more detail below.

###  Visualizing Pathways and Overall System Architecture

**Figure 3:**
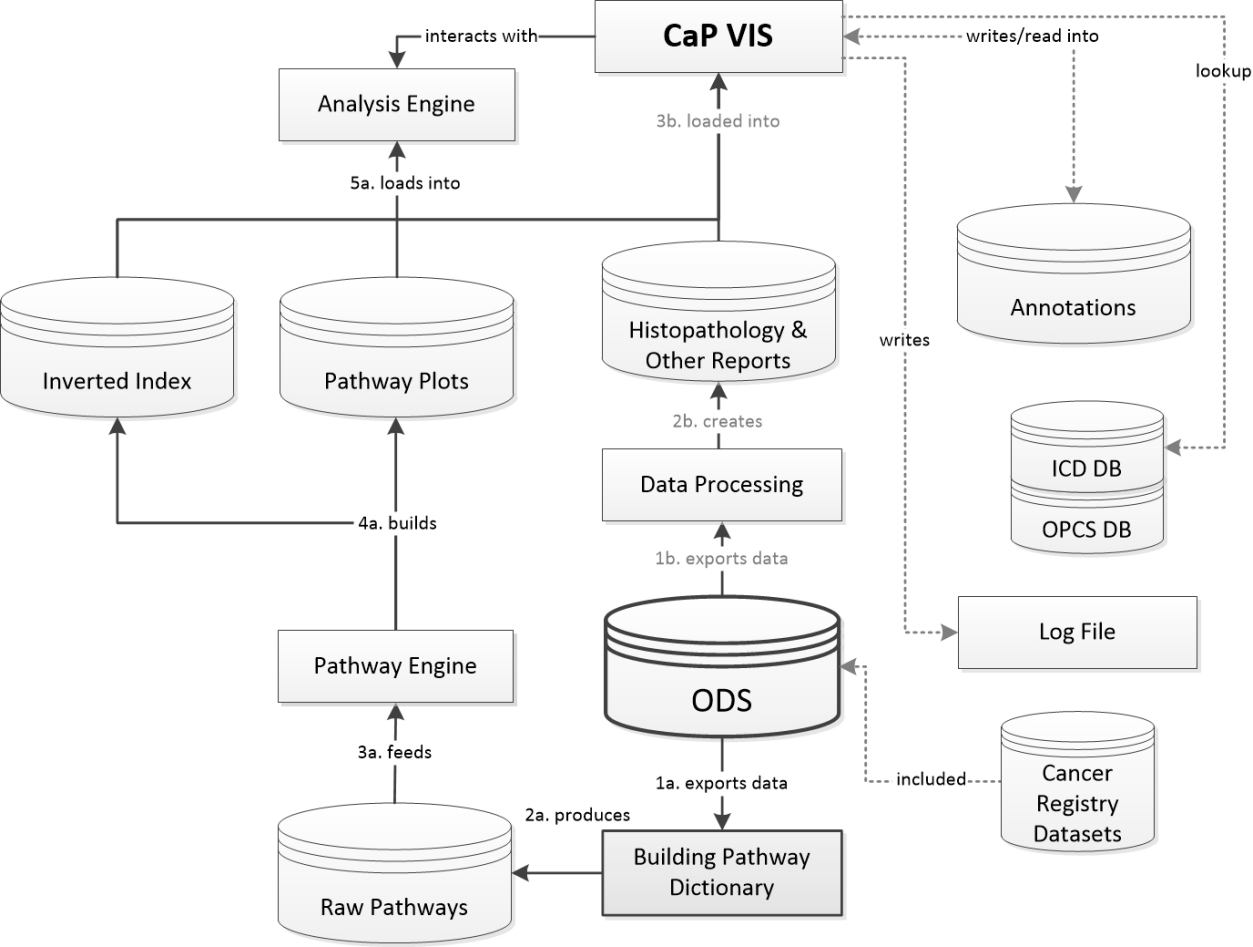
Data flow diagram illustrating the relationship between the operational data store (ODS, in bold), the pathway and analysis engine, the carcinoma of the prostate visualization and interpretation system (CaP VIS), and other interactions including lookup databases (DB) for International Classification of Diseases (ICD) and OPCS (Office of Population Censuses and Surveys) coding. The two main processes that feed information into the CaP VIS system are enumerated. The pathways data follows steps 1a to 5a, while other information follows steps 1b to 3b. Secondary processes are highlighted with dotted lines.

A system responsible for the integration, visualization, and analysis of pathways and related data was developed. Figure 3 illustrates the overall environment of the developed carcinoma of the prostate visualization and interpretation system (CaP VIS), the ODS, and the previous method of building the pathway dictionary. Figure 3 also shows the ways in which the data flow from the sources, and the steps involved in bringing detailed pathways into the visualization and interpretation system, the analysis, or query engines. The steps of the two main processes that feed data into the CaP VIS system start from the ODS and are enumerated. Secondary processes are highlighted with dotted lines.

**Figure 4:**
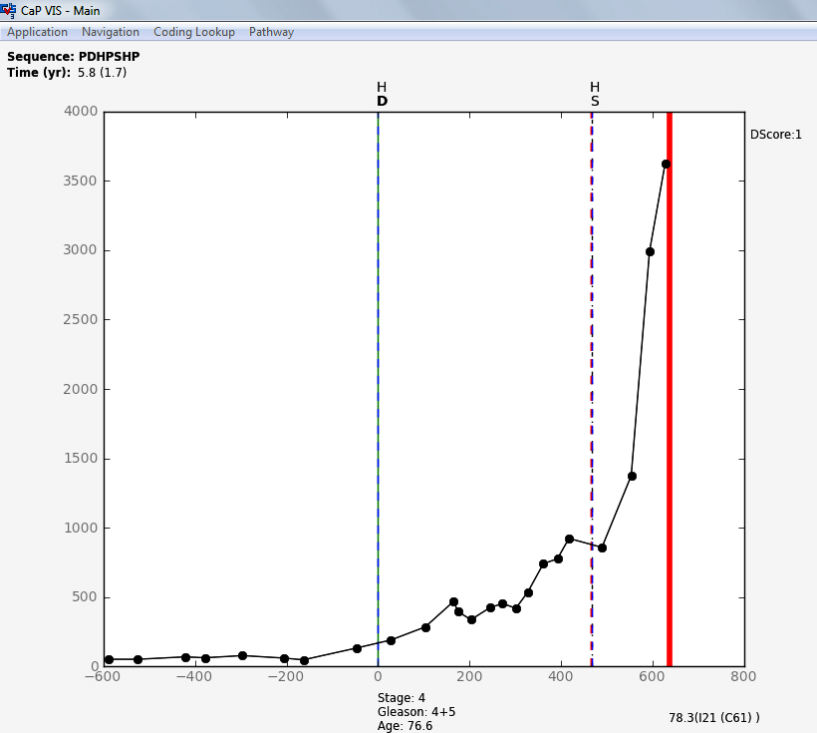
The CaP VIS system showing the left side of the screen with the graphical representation of a castration resistant patient pathway. The patient was first treated with hormone therapy and had a subsequent palliative prostatic resection. The plotted pathway shows the trend of the PSA biomarker together with diagnosis line and treatments.

**Figure 5:**
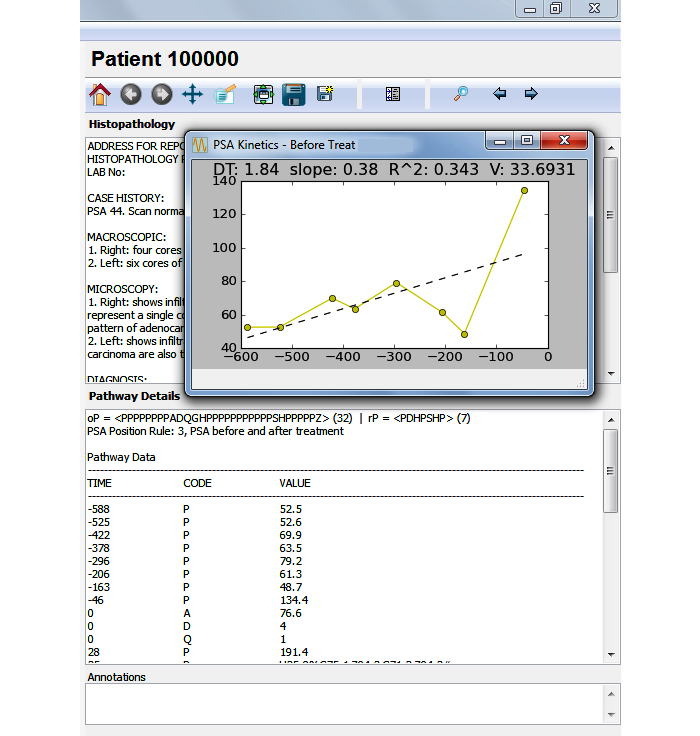
The carcinoma of the prostate visualization and interpretation system (CaP VIS) system showing the right side of the screen where additional information about the patient pathway is seen. There are three sections that show the histopathology report, the pathway details in the format presented in this paper, the annotations section, and a fourth section (hidden) contains pathway statistics. An overlapping window shows in detail the prostate specific antigen (PSA) kinetics before treatment, computed for this pathway. The toolbar above the histopathology section allows the user to zoom and pan the plotted pathway (in Figure 4), as well as to save the plotted figure to file, search for particular patient pathway, or navigate to the previous or next patient pathway. Doubling time (DT) and velocity (V).

The main process responsible for producing the pathways starts from the ODS and follows steps 1a to 5a in Figure 3. Datasets were extracted from the ODS in the pathway format defined in the subsection “Defining a Pathway”, and used to build the pathway dictionary (as described in the subsection “Building the Pathway Dictionary and Database”), and the raw pathways database (following steps 1a and 2a). The pathways engine, which works with the information stored in the raw pathways database (step 3a), is responsible for the segmentation, summarization, cleansing, and indexing of the raw pathways. Such operations together allow for the mapping, selection, and retrieval of individual or groups of similar paths using regular expressions or ad-hoc algorithms. The detailed pathways are organized by patient identifier and stored as “plots” (following step 4a) that allow an interpreter and the visualization software (CaP VIS) to produce a detailed graphical representation (step 5a). The interpreter will parse each activity from a pathway and, based on the dictionary and a set of rules determined for each element code, plot the corresponding graphical representation. An important feature of the visualization system is to integrate the pathways with histopathological or further clinical information. A coding lookup table was added in order to translate and present diagnosis (International Classification of Diseases, ICD) and procedures (Office of Population Censuses and Surveys) codes (highlighted by the dotted lines in Figure 3). Because the time length of different pathways can vary considerably, it was important for the plot to be interactive, allowing zoom and rescale, as well as mechanisms for graphical conflict resolution (ie, avoiding overlapping elements). Figures 4 and 5 show sample output from the visualization software and a patient pathway and related information, including the pathway data format. The analysis engine can be used by the CaP VIS software to compute statistics for the pathways, but it can also be used on its own to develop algorithms that work with the pathways data. The subsection “Assessing Completeness Using Biomarker Information” demonstrates the use of the analysis engine in computing completeness scores for the PSA values in pathways. The analysis engine consists of a set of functions and libraries that are built in to main software, written in Python. In order to access the engine and perform operations, Python scripts can be written to access relevant functions that read information from pathways, generate graphical representations, compute PSA kinetics, or other statistics.

The CaP VIS system is also fed additional data that can be linked with pathway information. This process follows steps 1b to 3b in Figure 3, and produces a database of other clinical information not included in the pathways dictionary, such as full histopathological text reports. The latter could still be included in the pathways, but in our case study, this information was not part of the desired graphical representation of a pathway, and so it was more efficient for it to be accessed differently. Furthermore, this enables the system to use additional data that are not part of the pathway. The CaP VIS system integrates this information and shows a novel graphical representation of the patients' pathways. Figure 4 shows the left side of the CaP VIS screen where the graphical representation of a pathway is visible, and Figure 5 shows the right side of the screen with additional information pertaining to that pathway. Together, the two figures show the full screen of the system.

The way in which pathway elements are plotted in CaP VIS depends upon their code, type, and value. In the pathway plot seen in Figure 4, the x-axis represents the time in days, zeroed on diagnosis of prostate cancer, and the y-axis represents the biochemical marker, PSA. Other data elements are plotted either as vertical lines dividing the pathway into segments, or as further information captions along the x-axis or y-axis, as needed. The plot illustrates a total of 32 events and informational elements. Vertical lines pertaining to treatments or diagnostics are accompanied by the respective element code from the dictionary on top. There are three types of vertical lines that are plotted: diagnosis (code, D, solid line), treatment (codes, H for hormone therapy and S for surgery, dashed), and death (bold). The latter is accompanied, along the x-axis, by ICD coding for the causes of death as well as age at death, whereas the diagnosis line is accompanied by age at diagnosis, tumor staging, and Gleason grade. Treatments are plotted as dashed lines and further biopsies, dotted lines. The lines may overlap; however, color coding and scaling are available to further investigate smaller segments of the pathway when necessary.

The main CaP VIS system screen contains three areas on the right side of the screen (Figure 5) to enable the inclusion of the histopathology text reports, pathway details, and annotations. The histopathology box can be toggled between a summary of the pathway statistics including PSA kinetics (measurements of change over time, widely used to assess recurrence) [28] or the histopathology text report. The pathway details box includes the pathway data in the format described in the subsection “Defining a Pathway”, and further information computed based on that data. Other screens include the detailed PSA kinetics regression line (seen in Figure 5 above histopathology text report, including doubling time and velocity), and a further screen (not shown here) summarizing the details of all 1904 pathways, which can be used to query the cohort. Overall, the CaP VIS system allows a thorough inspection of biomarker trends and other electronically available data on patients with prostate cancer by clinicians and researchers.

###  Assessing Completeness Using Biomarker Information

Routinely collected data can vary in quality, and it is important to assert the quality of the elements in the pathway so that they can be selected or discarded for clinical analysis. The above sections dealt with the definition and building of pathways from routine data, and this section introduces a method for inspecting their quality with the aim of selecting pathways for clinical studies. We already discussed that upon extraction from the ODS, data elements were previously cross-validated against a trusted source, namely, the CR. However, the biomarker information available in hospitals and included in this study enabled additional quality assurance. To this effect, we investigate methods of computing the completeness of pathways from the biomarker information. For this, rule-based scores were computed. The data elements used to assess pathway quality were the PSA and all radical treatments available in the pathway dictionary (ie, treatments that have an impact on the biomarker). The reason for choosing these elements is their interest for the analysis of prostate cancer, and hence, their ability to indicate the quality of the data for that specific purpose. Clinicians are also interested in biomarker trends and in comparing patients under different treatment regimes. Similar methods may be applied to other variables or domains, and should allow the assessment of data quality for different clinical investigations.

###  Rule-Based Scores

Given the defined dictionary and its underlying format, it is possible to create a knowledge base of rules to aid the process of computing completeness scores for particular elements of the pathway. It is often difficult to convey and analyze a biomarkers’ information in pathways, but here it was possible to compute their trends and to allow those computations to inform on the quality of the pathway. In the particular case of prostate cancer, the trend of PSA readings across the pathway is of interest. We identified, guided by domain experts, two major sets of rules in which the biomarker can be used to assess the completeness of a pathway with respect the clinical domain. The first set of rules relies on the position of biomarker readings in the pathway, whereas the second relies on identifying clinical interventions that justify abrupt changes in biomarker values. Rules can be applied programmatically by running Python scripts in the analysis engine. In this case study, the rules were used to help determine the quality of the pathways for future research. However, similar rules can be built to assess adherence to guidelines, or to perform complex data queries.

### Positioning of Biomarker Readings

As some of the intended clinical investigations pertain to PSA trends and associated treatments, it is important to have complete PSA trends within a pathway. In this context, a pathway should include biomarker readings before and after treatment so that the effect of treatment on the biomarker can be elucidated in posterior analyses. We can therefore compute a partial score of a pathway as a result of a set of rules on the occurrence of PSA readings. The rules are presented in Table 3 with their respective score and the percentage of pathways where the rule applied. The computation of the positioning score involves iterating through pathways’ codes and flagging occurrences of the PSA and their position with respect to treatments (excluding active surveillance). The most informative pathways should have one or more readings before and after treatment and the least informative have no PSA readings.

**Table 3:** PSA availability and positioning rules with respective scores and coverage.

Positioning score	Rule description	Coverage, n (%)
0	No PSA readings found.	90/1904 (4.73)
1	One or more readings found before treatment (or no treatment) and none after treatment.	77/1904 (4.04)
2	One or more readings found after treatment and none before treatment.	158/1904 (8.30)
3	One or more readings found before and after treatment.	1579/1904 (82.93)

### Substantiation of Biomarker Variation

Further rules can be devised to ascertain quality. For example, biological variations, in this case expressed by the PSA, should often be accompanied by evidence of some clinical intervention or other relevant factor. In the case of prostate cancer, an analysis of the PSA curve can be undertaken to identify major changes in PSA readings. In this case, the most significant drop in PSA should be associated with treatment to the prostate. A complete pathway for our purposes should attempt to provide explanations for such drops in the form of some clinical intervention. In this case, the computation of a score involves looking at every pair of PSA readings and then identifying the maximum absolute drop. Searching between the pair of readings to identify an element of substantiation, which in this case study was set to be any radical treatment, follows this. The result of this rule is a Boolean value, stating whether substantiation of a large change in the biomarker trend was detected. Although this rule may in most cases provide relevant insights on data quality for patients with prostate cancer, the use of other biomarker variations to inform on quality should be carried out with caution, as other potential factors could introduce bias. This has been discussed in detail in [29,30].

### Overall Score

An overall score for completeness can then be computed based on both positioning of biomarker readings and substantiation of major variation. It is worth noting that pathways that receive a positioning score of 0 or 1 could not have substantiation by definition, as no PSA values appear after treatment. The overall score is an ordered set of values in which the highest score is awarded to the pathways with the highest positioning scores that are substantiated. The overall scores are exemplified in Table 4 (see Multimedia Appendix 1).

**Table 4:** Completeness scoring system for PSA trends in prostate cancer pathways.

Overall score	Biomarker			Frequency, n (%)	Average number of unique elements
	Positioning	Substantiation	Description		
S0	0	N/A^a^	No readings found.	90/1904 (4.73)	3.26 (SD .64)
S1	1	N/A^a^	One or more readings found before treatment (or no treatment), and no readings after.	77/1904 (4.04)	4.72 (SD 1.02)
S2	2	N/A^a^	One or more readings found after treatment, and no readings before.	102/1904 (5.36)	4.71 (SD 1.03)
S3	3	No	One or more readings found before and after treatment.	393/1904 (20.64)	4.56 (SD .99)
S4	2	Yes	One or more readings found after treatment and major biomarker variation explained.	56/1904 (2.94)	4.70 (SD .88)
S5	3	Yes	One or more readings found before and after treatment and major biomarker variation explained.	1186/1904 (62.29)	4.80 (SD .92)

^a^N/A=not applicable

##  Results

### Building Pathways

The development of a framework to build, analyze, and visualize pathways from routinely collected hospital data made it possible to create individual patient pathways for 1904 patients, while integrating clinical information from several HIS.

The developed data dictionary contains 16 elements, described in Table 2. The data sources specify whether the elements were collected from the ODS (hospital systems, together with an abbreviation of the respective system) or the CR. Elements present on both sources have been cross-validated so their quality is assured. The quality and accuracy of the data elements present in the pathways was ensured in the process of building the pathway dictionary. Quality checks were also performed when building the ODS, and additional clerical review was undertaken manually.

Table 2 shows the element’s frequency, and indicates the percentage of pathways in which that particular element is present. Table 2 also gives the percentage of patients who died in this cohort during the time of observation (ie, pathways including a death event, 21.11%, 402/1904). These deaths are not exclusive to prostate cancer, and the percentage should not be used to determine a measure of survival from prostate cancer. It will be possible, however, to undertake survival analyses in future studies.

Regarding biopsies, they are only coded if performed as an inpatient event, and hence, only extensive biopsies were retrieved. As a result, biopsy events were removed from the dictionary and are not used in the current study, but can be kept for future studies. The frequency of imaging events was low (only captured imaging events on 15.28%, 291/1904, of all pathways), and it reflects the nature of the retrieval methods from radiology, which are based on a text search of the word “prostate”. Further data elements that have not been added to the dictionary here, but will be added in future studies, include further biochemistry tests as well as comorbidities and hospital stays, which may or may not be related to prostate cancer.

The analysis engine computed descriptive statistics, such as the various frequencies of the elements of the dictionary. A summary of the pathway statistics for all pathways is given in Table 5. Descriptive statistics are important as they convey information about the pathways. They can also give rise to quality indicators, but we found these methods alone not to be sufficient to determine quality.

The use of routinely collected hospital data for timed events indicates with certainty that a particular activity occurred; however, its absence may not indicate the opposite. Existing data may be used in validity checks for the completeness of the data, for example, the PSA biomarker can act as an alert for potential missing activities at particular time intervals. The pathways’ data structure and analysis engine enabled the computation of completeness scores for the purpose of selecting pathways with similar data points to analyze the biomarker trend. The analysis engine allows other rules to be implemented, including measuring the time between PSA measurements, for example. The following sections show the results of the application of the rules and their impact on quality assessment for research purposes.

**Table 5:** Summary of pathway statistics.

Statistic	Value
Average number of unique activities	4.66 (SD 1.03)
Average pathway length	1795 days (SD 1724)
Average pathway length from diagnosis	1017 days (SD 653)
Most common activity code, n (%)	P 1723/1904 (90.49)
Five most common start codes, n (%)	P 1388/1904 (72.89) X 222/1904 (11.65) D 141/1904 (7.40) G 79/1904 (4.14) L 22/1904 (1.15)
Five most common terminal codes, n (%)	P 1394/1904 (73.21) Z 399/1904 (20.95) G 59/1904 (3.09) W 12/1904 (0.63) R 10/1904 (0.52)
Total number of unique pathway sequences	694
Most common pathways’ sequence (repetitions truncated), n (%)	{P,D,G,H,P} 135/1904 (7.09) {P,D,G,W,P} 130/1904 (6.82)
Most common treatment regimes (where first and second treatment modality are within 92 days of each other), n (%)	H^a^ 907/1904 (47.63) S^b^ 518/1904 (27.20) W^c^ 318/1904 (16.70) SW^d^ 59/1904 (3.09) SH^e^ 22/1904 (1.15)

^a^H=hormone therapy alone ^b^S=surgery alone ^c^W=watchful waiting alone ^d^SW = surgery and watchful waiting within 92 days ^e^SH = surgery and hormone therapy within 92 days

### Inspection of the Positioning of Readings

The application of rules on the positioning of the PSA biomarker allowed the identification of (82.93%) 1579/1904 pathways, where it was possible to plot the trend of the biomarker through treatment (scores S3+S5 in Table 4). The framework presented above made possible the inspection of data elements in relation to other events plotted chronologically. It is also possible to compute the proximity between elements. For example, treatment elements within 90 days were grouped together to form treatment packages. The type of rules proposed here allow for the assessment of the timeliness and completeness dimensions of data quality.

### Inspection of the Substantiation Rule

Overall, it was possible to ascertain the biomarker variation substantiation rule for 61.08% (1163/1904) of the pathways. We also identified that 4.14% (79/1904) of pathways with two or more PSA readings had a constant or always rising PSA trend. These were merged with the overall substantiation number, making 65.23% (1242/1904) the total number of pathways with a positive substantiation rule (scores S4+S5 in Table 4). Substantiation does not occur when a treatment element is not present in the biomarker interval of interest, or if the treatment date is inaccurate. This may indicate missing information in the case of prostate cancer. The substantiation rule allows for the elimination of pathways with insufficiently accurate information to study the biomarker trend. This rule enables the assessment of completeness and timeliness dimensions of data quality. However, it should be used with caution, in particular in other domains, as other factors may also explain the variations in the biomarker trends.

### A Hybrid Scoring System

A hybrid scoring system for the completeness of the pathways combines both biomarker rules described above (positioning and substantiation), and it is given in Table 4 and examples are given in the Appendix (see Multimedia Appendix 1). The overall score ranges from least complete (score S0) to most complete (score S5), and were automatically computed based on the criteria set in the rules above. Example pathways for each computed score are given in the Appendix (see Multimedia Appendix 1). This particular set of rules aims to identify the completeness of the pathways based on the prostate cancer biomarker. It is also possible to extend the framework presented in this paper to create other quality scenarios involving more robust and detailed rules based on biomarkers or other aspects of the pathways. Examples of pathway plots automatically drawn by the CaP VIS system are available in the Appendix (see Multimedia Appendix 1) and illustrate each of the five completeness scores.

### Further Analysis of Data Quality

A further analysis on surveillance regimes made possible the observation that 7.3% (25/342) of those on surveillance (as first treatment) had a subsequent treatment within at least a year, and therefore left surveillance. For those that did not have a subsequent treatment (92.6%, 317/342), it was possible to investigate any substantial drops in PSA, which may be indicative of unrecorded treatments. By establishing a drop ratio calculated as the maximum PSA drop divided by the PSA at diagnosis, we noted that 30.6% (97/317) of pathways on surveillance regimes show a drop over a 0.5 ratio, whereas 15.7% (50/317) had a drop >1. This analysis is only preliminary, but it may indicate that patients received treatment, yet these have not been recorded or carried out at this hospital. Such pathways could be excluded from analyses or be further explored to seek plausible reasons for the unexplained variation in biomarker trend. Again, this is an example of the type of analysis enabled by the framework and the pathways’ data structure.

The analyses on quality also led to improvements in the data collection process. It was possible, for example, to identify patients that only had PSA readings after treatment, as well as those without PSA readings before diagnosis. This process yielded a small number of pathways (2.00%, 38/1904) where there had been earlier PSA readings, but these were not linked to the patient’s main hospital number in the hospital data warehouses, and hence, were missed on retrieval (not present in the ODS). Such cases are not expected to occur frequently, and do not affect any of the hospital administration or clinical operations. However, they can diminish the amount of information available for the use of routinely collected hospital data for analysis. In this instance, as only a small number of cases were affected, they were manually fixed. The exercise, however, uncovered the need for further checks by the hospital on the data warehouse to ensure consistency of recordings.

### Framework, Developed Software, and Visualization

**Figure 6:**
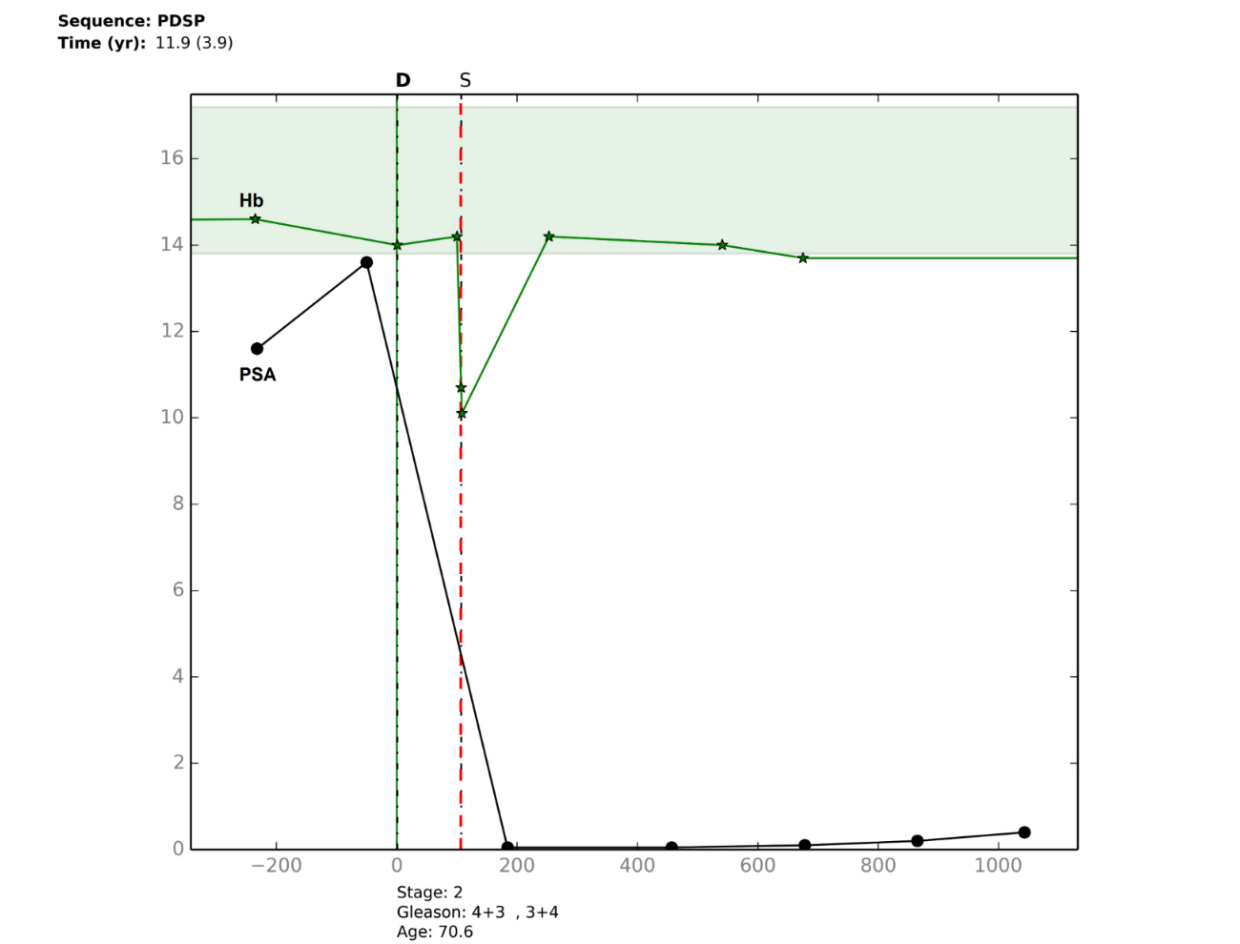
Pathway plot showing the prostate specific antigen (PSA) (round markers) and haemoglobin (Hb) readings (star markers) together. As a result of the prostatectomy event (S) the PSA dropped and Hb also dropped due to normal perioperative bleeding. The shaded area denotes the normal range for Hb.

The developed framework and visualization software enabled the visualization of all 1904 patient pathways with their corresponding biomarker trends. This gives clinicians access to trends that may have been previously much harder to observe. Furthermore, the system is flexible and extensible to include other data elements such as blood readings. For example, Figure 6 shows the PSA values and the haemoglobin (Hb) readings. The shaded area is the normal range for Hb. In this case, the drop in Hb on the day of surgery reveals perioperative bleeding. This information, when computed for all patients, would enable a study of the length of time that patients take to recover after surgery. This illustrates the flexibility of the combined framework and visualization tool, and provides access to a number of studies with data that was otherwise not readily available or contextualized. Furthermore, by plotting this, clinicians are able to see the full profile of the patient with respect to diagnosis, treatments, and how these affect the biomarker and other blood values. The pathways dictionary can continue to be developed to introduce additional information to this graphical representation.

This work contrasts with other established summarization and visualization systems, such as LifeLines [31], HARVEST [32], and others [33,34], in that it provides a succinct graphical and temporal representation that enables clinicians to promptly read a large number of data points and their interactions for a given patient in a single graph. However, this approach was developed to work with a single clinical domain of interest, while other systems may cope with multiple or overlapping domains and more complex data interactions, thereby summarizing larger amounts of information from EMRs. Nevertheless, the overall software and framework are also capable of handling the temporal complexity of constantly changing variables and producing unique meaningful visualizations for clinicians and other scientists.

Additionally, the framework presented made possible the inspection of data quality dimensions similar to those described in [11], including those that are least often assessed. The inspection of some of the dimensions, however, depends on the availability of the data elements in the sources. Currency (or timeliness) has been considered a fundamental dimension, yet it is often not assessed and only measured using a single approach [11]. The pathway data structure presented here includes time as one of the key variables, hence, it allows for the examination of currency; pathways are arranged chronologically and allow for concurrent elements. For example, in the case study, treatments within 90 days were considered as a treatment package. Another example of currency evaluation is the identification and discarding of data elements not relevant at particular intervals, as exemplified by the positioning rules. Furthermore, the plausibility and concordance dimensions were assessed with respect to PSA using the substantiation rule, the completeness dimension using the positioning rule, and correctness and completeness dimensions assessed by cross-referencing against the CR. The methods used correspond to log review (currency); validity checks (plausibility and correctness); element presence (completeness) and agreement (concordance); data source agreement; and gold standard (completeness and correctness).

The proposed framework and developed software should also allow for the selection and extraction of particular datasets with complete data for process mining and other analysis. It has been reported that the evaluation of the quality of process mining event logs relies on trustworthiness (recorded events actually happened), completeness, and well defined semantics [35]. These can be achieved by selecting pathways with required data points using the proposed framework. Furthermore, the visualization system allows for the close inspection and contextualization of pathways, illustrating particular paths with similar features, such as the ones exemplified in the Appendix (see Multimedia Appendix 1). In summary, the proposed framework, when used in hospitals, would facilitate the retrieval, selection, and inspection of patient pathways, and also the further steps of data mining analysis using appropriate methodologies.

##  Discussion

### Principal Findings

Based on the prostate cancer case study carried out at a large regional NHS hospital, a framework, which enables the secondary uses of routinely collected hospital data, was developed and presented in this paper. The main components of this framework (Figure 3) are the ODS containing patient-centric data, used to build the pathways based on the methodology presented in Figure 2; the pathways engine; analysis engine; and the visualization software. The underlying pathway data structure, in some aspects similar to the EAV data model, retains some degree of patient privacy and together with the dictionary provides a simplified, yet flexible and powerful, platform for the complex querying and analysis of patient information and disease pathways. It enables the summarization and extension of pathways, as well as the aggregation of similar sequences. It is also possible to capture and plot pathways with concurrent elements, and to develop algorithms to further explore the data and investigate quality issues. Furthermore, the methodology used to build the pathway dictionary, as well as the formalisms presented here, can be transported to other domains and settings. This is particularly true because the pathways dictionary can be remodelled to accommodate other data elements and research interests. Likewise, the framework is capable of plotting other continuous or categorical variables. The software has also been developed in a way that accommodates changes, as it focuses on the pathway data model (subsection “Defining a Pathway”) that is not designed for a specific disease. Nevertheless, in this paper, the pathways were constructed using a case study on prostate cancer, and further work is underway to apply these methods to other domains, where the emphasis is on different clinical parameters.

The process of integrating routinely collected electronic data may produce pathways that may not be informative or complete. A topic, which, to our knowledge, has received little attention in the literature, is the computation of quality indicators for data-driven pathways. Such indicators are important to enable the selection of study-relevant high quality data for clinical investigation. The methods developed in this paper enable us to discard pathways that, because of the nature of electronically routinely collected hospital data, fail to provide enough or sufficiently accurate information to be used in clinical analyses.

We have shown that methods for pathway quality measurement can rely on biological marker trends, as they are often the response to some parallel process. In the case of the PSA, a sharp decline in the average readings would most likely indicate treatment to the prostate, which suppressed the production of the antigen. This allows us to ascertain whether treatment records are missing. Similar approaches, however, should be used carefully so as to take into account any possible confounding factors. Algorithms were written to compute completeness based on prostate cancer biomarker rules, creating an overall scoring system (Table 4). Once researchers are satisfied that the PSA trends have sufficient data points and are substantiated (ie, they receive a high completeness score), they can investigate those PSA trends as predictors of prognosis in the disease. Such research is seldom undertaken due to the unavailability of data, but may lead to improved outcomes for patients and health services.

We investigated the cohort of 1904 patients, automatically built their respective pathways, and computed completeness scores with regards to the prostate cancer biomarker. Overall, 65.23% (1242/1904) of pathways attained the two highest scores, while 82.93% (1579/1904) attained the highest PSA positioning rule. Hence, these pathways contain sufficient biomarker information to aid clinical investigations on the biomarker trends. We have shown that routinely collected data can be transformed and prepared for clinical research, decision making, and decision support.

The flexibility of the data structure allows the insertion and removal of dictionary elements, and work is underway to include additional blood tests and comorbidities to the pathways, as depicted in Figure 6. The work presented here has also enabled future research into pathway adherence and variance metrics, particularly with respect to the UK NICE guidelines. This work is possible in the first instance by analyzing similar pathway sequences, and then by programmatically accessing detailed pathway information using the analysis engine. This paper describes methods for data collection, presentation, and quality assessment that can be applicable to build other disease pathways in other settings. We are also motivated by further work on mining pathways, in particular, the computation of similarity of biomarker trends, and the application of clustering algorithms and survival analysis in the context of pathways.

### Limitations

The framework and pathways were built using a case study on prostate cancer where there was a particular clinical interest on the biomarker trends. This specific working domain may introduce some limitations to the reproducibility of this work; however, further research is underway to apply the approach to other domains, specifically in the construction of pathways for acute stroke.

The number of data elements used in the pathway data dictionary was also a limiting factor, however, they were sufficient to study the PSA trends and to select cohorts with similar baseline features for further research. The pathways data structure presented in this paper has coped with the addition of new data elements, but further work is required to assess the quality and availability of other routinely collected data. Further work on the methods for evaluating quality is also needed, and it is hoped that the adoption of systematic methods, such as those presented in this paper, encourages further research in this area.

With regards to privacy, the pathways data structure includes an anonymized patient identification number, replaces specific dates with time zeroed at diagnosis, and suppresses patient names, addresses, and postcodes. These have been sufficient to ensure the anonymity of the patients. However, it may be possible to utilize specific information to attempt to identify individuals, particularly as new data elements containing specific information are added. Further work may be required to anonymize additional information, such as histopathological text reports, and to ensure that the system is fully resistant to privacy attacks.

The timeliness of the process of retrieving routine data and feeding them into the pathways database depends on the availability of the data in the ODS, and it can be a limitation. Similarly, the process of building the pathways dictionary and liaising with domain experts may introduce delays. However, once the dictionary is agreed and the data and their sources are fully understood and accessible, creating individual pathways in near real-time is possible. In this case study, the process of transforming data from the ODS into the pathways database for a new data element could be achieved in a few hours, however, the retrieval of the data from the sources onto the ODS and liaison with domain experts and other hospital staff could introduce significant delays up to several weeks. This case study was also undertaken in a single large hospital and, although the challenges are reportedly similar elsewhere, it is expected that the time and effort to feed new routine data can vary considerably.

## Appendix

Multimedia Appendix 1 Examples of pathway plots drawn by the developed CaP VIS system for each of the six possible completeness scores.

## Abbreviations

CaP VIS: carcinoma of the prostate visualization and interpretation system
CR: cancer registry
CSV: comma separated file
EAV: entity-attribute-value
EHR: electronic health records
Hb: Haemoglobin
HIS: hospital information systems
ICD: International Classification of Diseases
LAB: laboratory information system
NHS: National Health Service
NNUH: Norfolk & Norwich University Hospital
NICE: National Institute for Clinical Excellence
ODS: operational data store
PSA: prostate specific antigen
