# Supplementary figures and images for: Building Data-Driven Pathways From Routinely Collected Hospital Data: A Case Study on Prostate Cancer
Source: JMIR Med Inform. 2015 Jul 10;3(3):e26. doi: 10.2196/medinform.4221 (PMC4526987; doi:10.2196/medinform.4221)

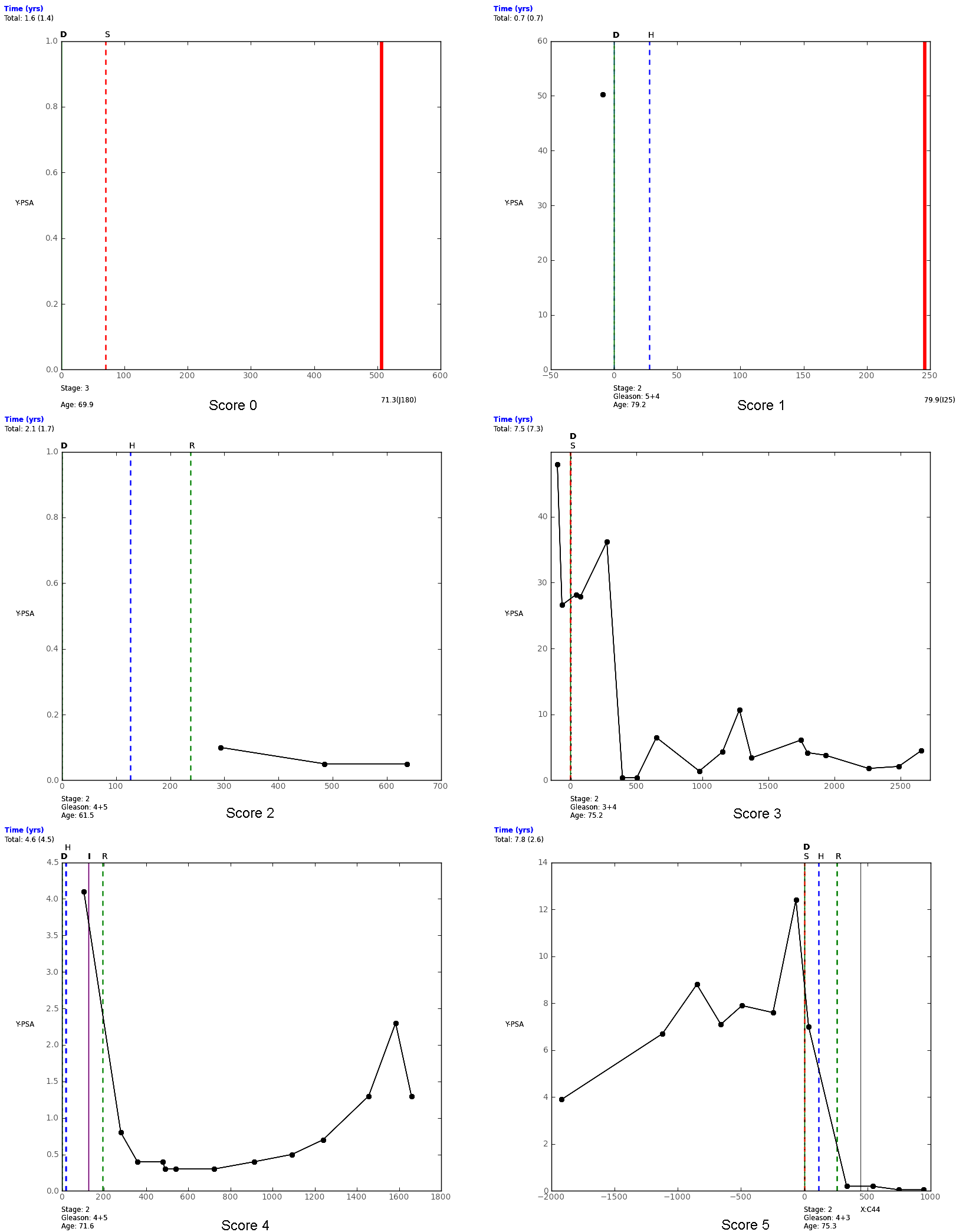

Supplement: Multimedia Appendix 1 [file medinform_v3i3e26_app1.tif]
